# Linear Elastic FE-Analysis of Porous, Laser Welded, Heat Treatable, Aluminium High Pressure Die Castings Based on X-Ray Computed Tomography Data

**DOI:** 10.3390/ma13061420

**Published:** 2020-03-20

**Authors:** Fabian Teichmann, Arne Ziemer, Martin Leitner, Jonas Hensel, Klaus Dilger

**Affiliations:** 1 Institute of Joining and Welding, Technische Universität Braunschweig, Langer Kamp 8, D-38106 Braunschweig, Germany; a.ziemer@tu-braunschweig.de (A.Z.); j.hensel@tu-braunschweig.de (J.H.); k.dilger@tu-braunschweig.de (K.D.); 2 Montanuniversität Loeben, Department Product Engineering, Chair of Mechanical Engineering, Franz-Josef-Strasse 18, 8700 Leoben, Austria; martin.leitner@unileoben.ac.at

**Keywords:** laser welding, high pressure aluminium die casting, aluminium, AC-AlSi10MnMg, X-ray computed tomography, finite elements, mesh, structural simulation

## Abstract

The welding of aluminium high pressure die castings is a well known and broadly investigated challenge in various fields of industry and research. Prior research in this specific field mainly focused on the optimisation of the welding and the casting process and on the cause of the frequently occurring porosity and incomplete fusion phenomena, whereas the impacts of these defects have hardly been addressed. Therefore, the underlying study presents the investigation of weldments in EN AC-AlSi10MnMg high pressure aluminium die castings by linear elastic finite element analysis based on X-ray computed tomography as a novel approach. Hereby, four laser weldments with differing surfaces and pore contents were investigated by X-ray computed tomography and tensile testing. Based on the voxel datasets of the porous weldments, triangular finite element meshes were generated and a numerical finite element analysis was conducted. Good agreement of the stress–strain curves between the simulations and the experiments was achieved.

## 1. Introduction

### 1.1. Defects in Laser Welded Aluminium High Pressure Die Castings

The occurrence of weld bead porosity and incomplete fusion are well known issues when welding aluminium, high pressure die casting (HPDC) alloys [1,2]. According to the German Welding Society [3], the cause of the porosity can be distinguished in metallurgical and mechanical pore formation. In the case of metallurgically formed pores, hydrogen is released into the melt, e.g., by the dissociation of aluminium hydrides, agglomerates within the melt pool and is trapped in the weld metal during the solidification of the melt. In the case of mechanical pore formation, inclusions or pores present within the die casting part are heated rapidly by the laser beam during the welding process. The heat input causes a rapid expansion of the pores, which leads to pore formation in the weld metal, an ejection of the molten metal and/or incomplete fusion. Thus, the weldability of high pressure aluminium die castings depends, amongst other factors, on the presence of inclusions and the hydrogen content [4].

Many studies have aimed to reduce the weld bead porosity and incomplete fusion. Thereby, measures considering the welding process were taken, as were those focused on the casting process. In terms of an optimisation of the casting process, Wiesner [4] stated that a sparse use of wax free, low concentrated lubricants and release agents has a favourable effect on the welding outcome. Moreover the application of vacuum high pressure die casting is a state of the art measure to positively influence the number of inclusions and the hydrogen content in a die casting part [2]. This, subsequently, enhances the weldability as well. Considering beam welding processes, many approaches were taken to optimise the welding outcome. Winkler [5] established the application of dual beam laser welding as an advantageous method to reduce porosity and to enhance the mechanical properties of the welded probes. Dittrich et al. [6] introduced laser welding with high frequency beam deflection as a method with which to feasibly achieve dissimilar welds of aluminium cast and aluminium wrought alloys. Fritzsche et al. [7] investigated the electromagnetic degassing of aluminium die castings during laser beam welding and found a positive influence of the electromagnetic forces on the welding outcome. In addition, the authors of the current study confirmed in prior research that dual beam laser welding has a positive impact on the weld bead quality [8,9]. In the same research it was found that the ambient pressure during laser welding had a strong influence on the amount, size and shape of the occurring weld metal porosity. Krahn et al. [10] reported a generally positive influence of multi spot electron beam welding on the occurrence of welding defects. These findings were further investigated and confirmed [11]. Despite the large variety of research carried out on the welding of aluminium high pressure die castings, the influence of the typically occurring welding defects on tensile strength and fatigue has not been investigated in detail.

Aside from aluminium HPDC alloys different research on laser vacuum welding of a broad variety of materials have been carried out in the past [12]. Experimental research has shown that the vacuum has several influences on the welding process and its outcome: For example, the increase of penetration depth, the degeneration of the vapour plume and the decrease of weld spatter were shown by diffrent authors [13,14,15,16]. In addition, Luo et al. [17] achieved the effects mentioned by the application of a local vacuum chamber during laser welding. Moreover, these observations were proven by numerical simulations of the welding process of Pang et al. [18]. Detailed investigations on the formation of porosity during laser vacuum welding of steel and aluminium have been carried out by Jiang et al. [19]. Jiang identified that the liquid metal flow around the keyhole changes its direction to an upward flow behind the keyhole. This upward flow accelerates gas bubbles in the melt and hence improves degassing.

### 1.2. Voxel-Based Numerical Simulation

Voxel-based numerical simulations have been carried out in different studies for various purposes [20,21,22,23,24,25,26,27,28,29,30]. Therein, different approaches were taken, such as the generation of tetrahedral meshes from the voxel data, the calculation on the voxel grids using the boundary element method and the boolean subtraction of extracted defects from virtual test specimens.

The basic idea, to utilise voxel data generated by X-ray computed tomography for numerical simulations, was already investigated in the health sciences during the 90s; for example, in [20]. Bossart et al. [20] demonstrated the tetrahedral volumetric mesh generation of a metacarpal bone. This approach was continued by others, such as Fang et al. [21], who investigated the tetrahedral mesh generation from volumetric images. The same approach was applied by Walle et al. [23]. In this study, the authors created tetrahedral meshes from XCT voxel data to predict the conductivity of porous sintered glass fibre material.

Nicoletto et al. [24] discussed the influence of casting pores on the fatigue behaviour of Al-Si alloys. The authors extracted a single pore from an X-ray computed tomography scan, embedded this pore as a void in various directions in a virtual tensile test specimen and investigated the impact of the pore on the fatigue behaviour. It was shown that the method is feasible for investigating the influence of complex 3D pore morphology on the fatigue behaviour. Wicke et al. [25] used the same approach and claimed the approach to be feasible for identifying stress concentrations.

Fieres et al. applied the voxel-based boundary element simulation method in [31] to predict stress accumulations in porous 3D printed parts and tensile test specimens made from aluminium. Based on the simulations, the authors were able to predict the stress distribution in the parts and test specimens, and the location of crack initiation and force necessary for the crack initiation. The presented approach is limited to elastic material behaviour and static loading. Despite these limitations, the results were accurate. Furthermore, Du Plessis et al. applied the same approach in [26] to investigate the impact of porosity in cast titanium Ti6Al4V on the tensile strength. The investigation of numerous test specimens using X-ray micro-computed tomography, tensile testing and numerical simulations, lead to the conclusion that the pores had an influence on the ductility and the location of failure. Moreover the authors were able to show that the simulation method is feasible for predicting both effects.

The influence of porosity on the fatigue behaviour of additively manufactured Ti6Al4V was investigated by Tammas-Williams et al. [27] under the use of voxel-based finite element simulation. The authors produced tensile test specimens by the use of electron beam melting, conducted an X-ray computed tomography analysis and performed interrupted fatigue tests. From the XCT voxel data they derived FE-meshes and simulated the fatigue loading. Using this method, the authors were able to visualise the stress distribution in the region of defects and to draw conclusions about the manufacturing process.

Serrano-Munoz et al. [28] performed a similar test series on the influence of casting defects on the fatigue life of A357-T6 cast aluminium alloy. Their investigations used synchrotron in situ fatigue testing and image-based finite element simulation to assess the effects of the shapes and sizes of casting defects on the fatigue life. Using this method, the authors proved that both affect the fatigue life.

Together, these studies indicate that the effects of defects in aluminium HPDC parts and additively manufactured parts from aluminium on fatigue and strength have been investigated by different authors. Moreover, these studies have shown X-ray computed tomography is a feasible method with which to obtain realistic data of a test specimen containing inner and outer defects. Based on this data, realistic finite element meshes can be obtained or factors for the fatigue assessment can be derived. Since this method has not been investigated for welded joints from aluminium HPDC alloy, the current research aims to apply X-ray computed tomography based finite element simulation to porous, laser welded, aluminium high pressure die castings. In the scope of the underlying study is the generation of triangular finite element meshes containing a large number of pores and outer defects, such as undercuts or incomplete fusion. As a long-term goal, the results of the current investigation are planned to be used as a basis for the assessment of the impacts of different defects on the fatigue behaviour.

## 2. Materials and Experimental Method

The current study aims to investigate welded aluminium high pressure die castings containing typical defects related to welding by the use of voxel-based finite element simulations. Therefore, four test welds were created by laser vacuum welding. Tensile test specimens were taken from the welds and an X-ray computed tomography (XCT) analysis was performed. Subsequently, the test specimens underwent a tensile test. Based on the voxel data of the XCT-analysis, numerical finite element simulations were carried out and compared to the experimental results of the tensile tests.

### 2.1. Materials

For the underlying investigation, aluminium high pressure die casting test plates of dimensions 260 × 150 × 4 mm^3^ were casted on a BüHLER EVOLUTION B 53D HPDC machine. The castings were produced by vacuum high pressure die casting with the use of rotor degassing to reduce the density index, and subsequently, the hydrogen content. The density index is a quality measure for gas remaining entrapped within the casted aluminium part and correlates strongly to the hydrogen content, as shown in [32]. During the casting process, the wax-free release agent *SL 1697 S*, manufactured by CHEMTREND, was applied. The resulting test plates were casted from the alloy EN AC-AlSi10MnMg with a density index of 0.8%.

### 2.2. Laser Vacuum Welding

As described above, the reduction of the ambient pressure during laser beam welding has a significant influence on the welding outcome. When welding high pressure aluminium die castings, the ambient pressure present during welding has a strong impact on the amount, size and shape of the occurring porosity. Thus, numerous laser welding trials were performed at different ambient pressure levels of 100 hPa and 0.1 hPa to create a wide variety of welding outcomes. The laser source applied was a Yb:YAG disk laser emitting continuous light of 1030 nm with a beam parameter product of 8 mm × mrad. The processing optics applied had a collimation of 200 mm and a focal length of 280 mm, resulting in a focal spot 280 μm in diameter. The focal position during welding was set to −2 mm for all the welds performed, and the welding velocity was fixed at 2 m/min. Since welding under sub atmospheric conditions increases the penetration depth, the laser power was reduced according to Table 1 to achieve a fully penetrating weld in 4 mm thick aluminium. All welding trials were repeated three times, and seven test specimens were taken from each weld bead. From the specimens created in the described manner, four representative test specimens containing typical defects were selected and analysed as described below.

### 2.3. X-ray Computed Tomography

**Figure 1:**
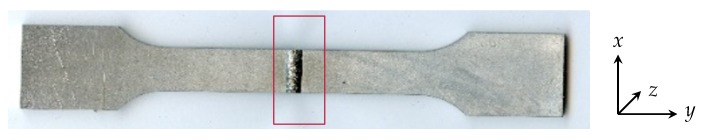
Welded tensile test specimen, area of XCT testing highlighted in red.

The X-ray computed tomography analysis was performed with a common commercially available scanner. For each scan, four tensile test specimens were mounted in a 3D printed polylactic jig. The XCT-scans were conducted under constant rotation of the specimens with 1442 images per scan. The exposure time was set to 100 ms, the tube voltage to 200 kV and the beam current to 180 μA. This setup resulted in a voxel size of 45.39 μm. The specimens selected for the current study were taken from a larger majority of samples, created for a previous study described in [33,34]. It has to be noted that the scanning parameters were predominantly optimised for time. The tensile testing specimens were XCT tested in the welded region only, as illustrated in Figure 1. The XCT-scans were reconstructed using the commercially available software *phoenix datos|X*. The porosity analysis was performed using the software *VGStudioMax 3.2*. From the porosity analysis, the number of pores for each weld bead and the sizes of the smallest and largest pores were extracted. Moreover, the mean sphericity of the pores detected within one weldment was calculated. Hereby, the sphericity is defined as the ratio of the surface of a perfect sphere with the same volume as the particle in consideration and the surface of the particle in question [35,36].

Within the XCT-scans, the coordinates are defined as follows: the x-axis points in the welding direction, the y-axis orthogonal to the welding direction and the z-axis in the direction of the penetration depth of the welding, as shown in Figure 5a. After the XCT-testing, the specimens underwent a tensile test.

### 2.4. Tensile Testing

**Figure 2:**
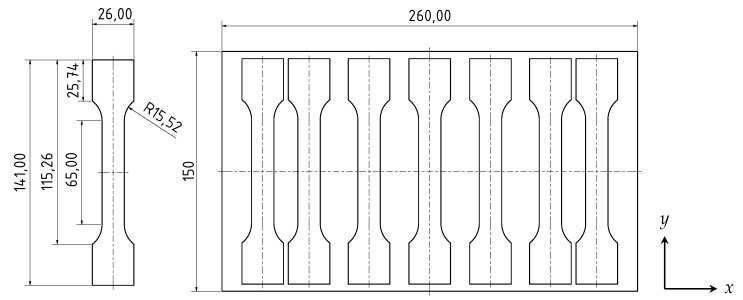
Geometry and location of extraction of the test specimens from the 4 mm thick high pressure die casting (HPDC) plate.

The weld beads were created in the middle of the die casting plates, as Figure 1 and Figure 2 indicate. From each weld, seven test specimens were taken by the use of waterjet cutting; see Figure 2. All test samples underwent a tensile test based on the DIN EN ISO 6892-1 [37] standard, using an INSTRON 5567 universal testing machine, consisting of a maximum test load of 30 kN. The tensile tests were carried out at a testing velocity of 1 mm/min; the strain was measured using an INSTON AVE 2663-821 Videoextensometer.

## 3. Numerical Method

The current study aims to investigate the feasibility of voxel-based structural finite element simulations of complex welded joints in high pressure aluminium die castings containing inner and outer defects related to welding. For this purpose, four datasets of XCT-data were chosen and submitted to the meshing algorithm described below. Afterwards, the meshes were used to conduct an elastic FE-Simulation of uniaxial loading. The results of the simulation were compared to the results of the experiments. In the current investigation, triangular meshes from differing sets of voxel data were created by the application of a custom-made algorithm using *MATLAB*.

### 3.1. Mesh Generation

**Figure 3:**
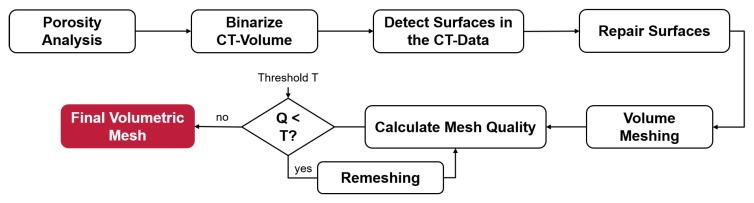
Flowchart of the proposed FE-meshing algorithm.

Figure 3 depicts a flowchart of the proposed algorithm for the finite element mesh generation: Firstly, the XCT-scan is reconstructed, registered and analysed for pores using *VGStudioMax*. Secondly, the resulting volume is binarised using a global threshold approach. Afterwards, surfaces within the volume data are calculated and repaired in a further step. Based on the resulting surfaces, the space between the surfaces of the body and the pores is meshed in C3D4 tetrahedra. The resulting mesh is then checked for Liu mesh quality [38] and remeshed in areas of insufficient quality in case a certain threshold T is not reached. In this research, a quality threshold of *T* = 0.5 was used. Once all bad elements were remeshed, the final mesh in the form of an *ABAQUS* input file was created. As mentioned above, the algorithm was programmed in MATLAB with the aid of the open source packages *ISO2MESH*, *DISTMESH*, *CGAL* and *TetGen* [21,39,40,41]. One advantage of the algorithm is that it allows for meshing each pore individually. Various processing steps, such as smoothing, mesh repair and refinement, can be added or neglected depending on the underlying dataset. Another advantage of this method is the ability to run it in parallel, since each pore can be processed on one CPU.

### 3.2. Finite Element Model

**Figure 4:**
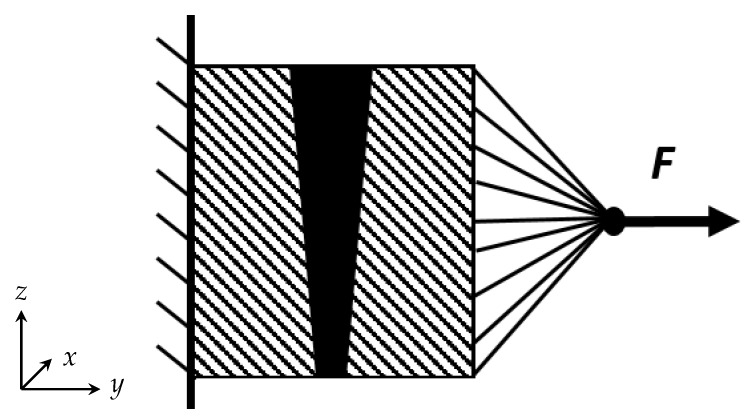
Cross sectional view on the geometry of the simulation model.

To simulate uniaxial loading on the welded samples, the finite element solver *ABAQUS 2016* was used. The simulated sections were cut from the XCT scans with a length of approximately 4 mm in loading direction, as depicted in Figure 1. In the finite element model a fixed support was added to the lower intersection of the simulated section. All nodes of the mesh located on the upper intersection were coupled to a reference node located approximately 5 mm above the intersection; see Figure 4. A force of 3 kN was applied to the reference node, which corresponds to an average stress of 62.5 MPa. In this research, it was aimed to describe the elastic behaviour. Thus, the simulation was conducted under the use of a Young’s modulus of 74,000 MPa and a Poisson’s ratio of 0.3 according to the literature [42,43,44].

## 4. Results and Discussion

### 4.1. X-ray Computed Tomography

**Figure 5:**
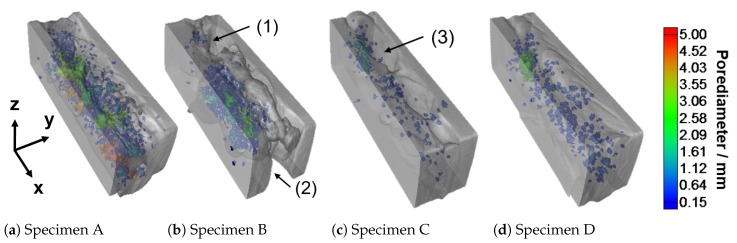
Grayscale volume models obtained from X-ray computed tomography showing incomplete fusion (1,2) and irregular weld surfaces (3). The pore diameter is depicted in pseudo colour.

Figure 5 illustrates the outcome of the X-ray computed tomography analysis of the selected weld seams. Herein, the pore diameter is depicted in pseudo colour. As seen in the images, the selected weldments contain a large variety of typical inner and outer welding defects. Regarding the surfaces of the welds, the specimens A and D have flat and regular surfaces, whereas specimens B and C show irregular weld surfaces; e.g., Figure 5c annotation (3). In addition, specimen B shows two areas of incomplete fusion; see Figure 5b annotations (1) and (2). In terms of the occurring weld bead porosity, the welds show either a small number of small pores (specimens C and D) or a large number of pores of differing size and pore nests (specimens A and B).

**Figure 6:**
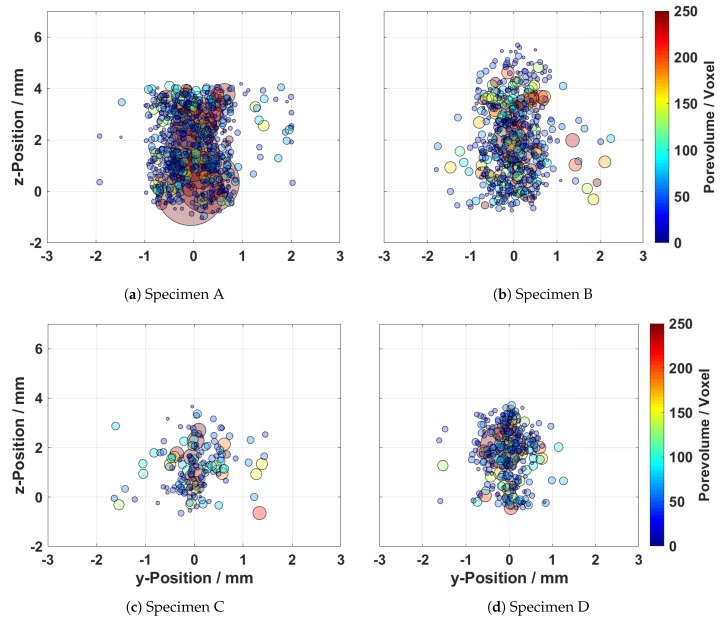
Cross sectional zy-projection of the position of the pores. The sizes of the pores are depicted in false-colours.

A projection of the position of each pore in the cross sectional view (z-y plane) of the weld is shown in Figure 6. In the diagrams, the sizes of the pores are illustrated in pseudo-colours. In general, the porosity is predominantly located in the centre of the welding in both the z and y-directions. Thus, the highest loss of material occurs in the centre of the welding.

Considering an approximated weld bead width of 2 mm, the plots also show pores in the region next to the weld bead. These pores are interpreted as base material porosity related to the casting process. It is assumed that these pores are a possible explanation for the melt ejections observed in Sample B. Welding in the region of casting pores causes a rapid expansion of the entrapped gases and the displacement of the molten material during the welding process.

Table 2 gives a detailed overview of the detected porosity present in the four investigated samples. This shows that Sample A contains the most and largest pores and the highest average of the cumulative volume of pores. Specimen B shows a lower number of pores and a lower maximum pore diameter compared to specimen A, and a significantly higher number of pores compared to specimens C and D. The lowest number of pores was present in weld C. Herein, it has to be noted that the load-bearing cross section of the weld is significantly reduced by the irregular surface and root of the weld (see Figure 8c). Specimen D showed a higher number of pores and a larger cumulative volume of pores compared to Sample C. As it can be seen from the Figure 5 and Figure 6, four welds of differing pore contents and surface appearances were created by the use of laser vacuum welding. Each combination of pore content and surface quality were investigated in the following by the procedure introduced in Section 2:

- Sample A: high pore content and regular surface appearance
- Sample B: high pore content and irregular surface appearance
- Sample C: low pore content and irregular surface appearance
- Sample D: low pore content and regular surface appearance

Based on the outcome of the XCT porosity analysis, it can be stated that the continuous CT-scanning method, in combination with the measurement of multiple specimens at the same time, successfully delivered voxel data sets of sufficient quality. The smallest pore diameter detected using the method and the parameters described above was 146 μm. Considering the applied welding process, it can be derived that welding under an ambient pressure of *p* = 0.1 hPa leads to an outcome showing a lower level of porosity than welding at *p* = 100 hPa.

### 4.2. Meshing of Voxel Data

Within the current research, data sets of four X-ray computed tomography scanned welding specimens were successfully meshed by the application of the proposed algorithm (see Section 3). Based on the properties of the created finite element meshes, it can be stated that the algorithm is capable of creating triangular meshes of an average Liu mesh quality of 0.74. The resulting meshes represent pores and undercuts, melt ejections or regions containing incomplete fusion, illustrated in Figure 5. In addition, the comparison of Table 2 and Table 3 shows that the algorithm can mesh all the pores detected by the porosity analysis.

From Table 3, it can be seen that the resulting meshes of specimens A and B contain approximately twice the elements compared to samples C and D. This results from the differing numbers of pores and outer welding defects, which enhance the number of surfaces, and consequently, the number of elements. In addition, the mesh in the region of the pores was found to be more fine; thus, there were more elements in that region. Consequently, this contributes to the increase of the number of elements for the samples containing larger numbers of pores.

### 4.3. Tensile Testing

**Figure 7:**
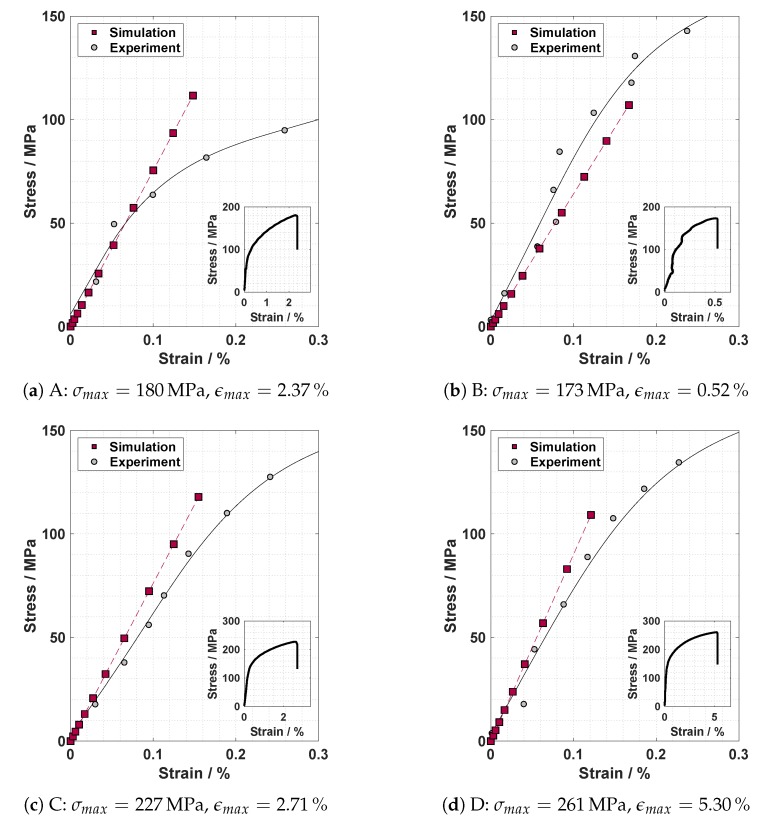
Comparison of the stress–strain curves for the welded test specimens from simulations and experiments.

The results of the tensile tests can be taken from the stress–strain curves of the welds illustrated in Figure 7. The stress was calculated based on the cross section of the tensile test samples (12 mm × 4 mm). Comparing the tensile tests, it has to be noted that specimens A and B were extracted from the same weld bead, and thus, were welded with the same parameters as specimens C and D (see Table 1). On that basis, and given the fact that the alloy used is heat treatable, it is assumed that the metallurgical condition of welds A and B was similar to that of specimens C and D.

As it can be derived from the figures, Sample A absorbed more strain at failure (2.37%) than Sample B (0.52%) at approximately the same maximum stress. It is almost certain that the reduced ability to compensate strains of Sample B is caused by the reduction of the load bearing cross section through pores and incomplete fusion; see Figure 5b annotation (1) and (2). In the case of specimens C and D it can be seen that specimen C absorbed 2.7% strain, whereas specimen D absorbed 5.3% strain. In addition, the ultimate tensile stress of specimen C is 34 MPa below that of specimen D. The observed discrepancies can be attributed to the differing appearances of the surfaces and roots of the weld beads. This is attributed to the fact that the porosity is approximately equal in both samples in relation to their load bearing sections (see Figure 5c,d). Thus, these observations confirm that both outer weld bead defects and pores have an influence on the strength of the weldment. In the case of the weldments discussed, an increased pore content (Sample B) had a stronger influence on the maximum stress and maximum elongation than an irregular surface (Sample C).

Furthermore, the comparison of the welds A and B with the welds C and D represents the general problem of the occurrence of large variations in the welding outcome when welding aluminium high pressure die castings. Samples A and B and the samples C and D were both taken from the same weld in the same material at varying positions; see Section 2.2. Despite these comparable circumstances, the welding results display strong variations in terms of appearance (e.g., Figure 5a,b) and mechanical properties. Therefore, an integral assessment of the welds, for example, by the use of X-ray computed tomography, is necessary.

### 4.4. Simulation

**Figure 8:**
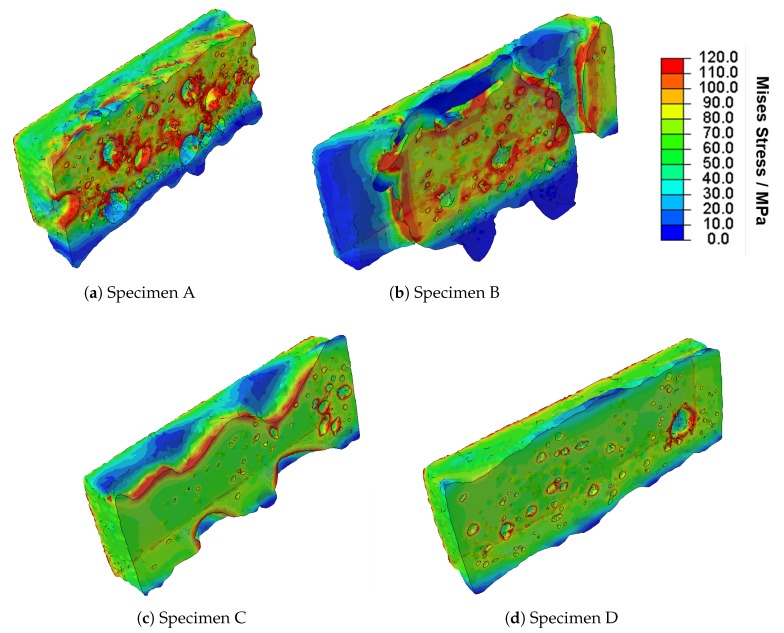
Results of the FE-simulation of the selected welds under a concentrated load of *F* = 3 kN; Mises stress shown in pseudo-colour. The welds are presented in the form of longitudinal cuts across the centre of the welds.

The results of the finite element simulations are illustrated in Figure 7 and Figure 8. Figure 7 compares the stresses and strains of the simulation and the tensile tests, where the stress is calculated based on the force applied to the reference node (compare Section 3.2). The plots depicted in Figure 7 show that the results of the numerical simulation fit to the experiments in the elastic region. The results revealed that the method applied is able to describe the material’s elastic response to uniaxial loading for porous welds (Sample A) with an irregular appearance of weld root and surface (Sample B). As depicted in Figure 7b, welds of an irregular appearance can be described by the use of the applied method. In the case of specimen A (see Figure 7a), the numerical results deviate from the experimental results when the stress exceeds 80 MPa. It seems possible that the heat input during welding caused a local decline of the yield limit, since the alloy used is heat treatable. Consequently, plastic deformation occurs in the experiments while the simulation still assumes linear elastic behaviour.

Figure 8 shows the von Mises stress distribution within the four test specimens when a force of *F* = 3 kN is applied at the reference node. The test samples are depicted in the form of longitudinal cutoffs through the centre of the weld. The pseudo-colours are illustrated from 0 to 120 MPa, where 120 MPa is a rough estimation of the base material yield strength at 0.2% in the as-casted condition [42]. Consequently, the regions displayed in red are interpreted as plastically deformed.

In all simulated geometries, stress concentrations can be observed in the region of inner and outer welding defects. These results reflect those of Fieres et al. [31], who found stress hot spots in the region of defects in additively manufactured Ti6Al4V parts; see Section 1. Comparing Figure 8a,b shows that there are more plastically deformed areas in Sample B than in Sample A. As it can be seen from the longitudinal cuts, the load bearing cross section in Sample B is reduced more than in Sample A, although there are fewer pores present. This may explain the reduction in maximum stress and elongation observed in the tensile tests; see Figure 7b. This hypothesis is supported by the comparison of specimens C and D, where Sample C shows larger plastically deformed regions than Sample D and a decrease in maximum stress and elongation. Moreover, it can be seen that the plastic deformation starts in the region of imperfections related to welding. The comparison of the four simulation results shows that a load of *F* = 3 kN has a stronger impact on the strongly damaged specimens A, B and C than on the slightly damaged Sample D. In addition, the simulations have shown that the method applied is strongly dependent on the voxel size. In the current study, the smallest pore which was detected had a diameter of 146 μm. Defects smaller than approximately four voxels are not implemented in the model. Considering smaller pores, in particular, those smaller than approximately 50 voxels, an accurate description of the shape is difficult due to the round shape of the pores and the cornered shape of the voxels.

## 5. Conclusions

The current research was undertaken to investigate voxel-based finite element simulation as an approach to visualising the influence of uniaxial loading on the stress distribution in the region of a welding defect. Therefore, four welding test specimens were created, and were characterised by the application of X-ray computed tomography and tensile testing. From the generated XCT voxel data sets, finite element meshes were created by the use of the proposed algorithm, and a structural finite element simulation model was created and computed. It was shown that the numerical model is able to visualise the stress distribution of the four welds of differing, complex geometrical appearances. Good agreement of the global stress and strain relationship between the experiment and simulation was achieved. It was demonstrated that it is possible to visualise the stress distributions in the regions of welding defects, such as pores, distorted weld surfaces and roots. In addition, the study showed that the simulation results fit to the outcome of the tensile testing during elastic material behaviour. Regarding the proposed algorithm, it can be stated that the generated meshes describe the test probe geometry integrally, including inner and outer defects.

Further research will cover the expansion of the created finite element model to predict the elastic-plastic material behaviour under uniaxial loading based on voxel data. Moreover the application of the presented method on the basis of XCT scans containing smaller voxels is planned. Further work will although be carried out on the validation of the strain fields obtained from the simulation by digital image correlation. On the basis of the findings of the current research, further work will further focus on the influences of differing defects, such as those investigated in this study, on fatigue behaviour.

## Figures and Tables

**Table 1:** Welding parameters of the selected test specimens.

Specimen	Laser Power	Ambient Pressure
–	W	hPa
A	2960	100
B	2960	100
C	2800	0.1
D	2800	0.1

**Table 2:** Results of the porosity analysis.

Index	Specimen A	Specimen B	Specimen C	Specimen D
Number of pores	708	544	148	298
Cumulative volume of pores/10^3^ Voxel	14.9	5.38	1.58	3.13
Minimum pore diameter/mm	0.14	0.14	0.15	0.15
Maximum pore diameter/mm	4.51	2.60	1.61	2.71
Median pore sphericity	0.59	0.59	0.57	0.57

**Table 3:** Properties of the created FE-Meshes.

Parameter	Specimen A	Specimen B	Specimen C	Specimen D
Number of Nodes	233,211	242,224	136,867	131,876
Number of Elements	1,052,983	1,069,364	581,831	567,605
Meshed Pores	708	544	148	298
